# Prepregnancy body mass index and risk of macrosomia after fresh embryo transfer: a retrospective cohort study with exploratory threshold analysis

**DOI:** 10.3389/fendo.2026.1884067

**Published:** 2026-07-15

**Authors:** Jing Wu, Ying Ju, Xiao He, Wanlin zhang, Fang Liu, Yuan Ma, Weiwei Kang, Man Di, Hengde Zhang, Jie Dong, Xiaohong Wang

**Affiliations:** Reproductive Medicine Center, Department of Obstetrics and Gynecology, Tangdu Hospital, Fourth Military Medical University, Xi’an, China

**Keywords:** birthweight, fresh embryo transfer, macrosomia, maternal body mass index (BMI), neonatal outcomes

## Abstract

**Background:**

Higher prepregnancy body mass index (BMI) is a well-established risk factor for adverse neonatal outcomes in IVF-conceived pregnancies; therefore, weight management is important for infertile patients, yet it remains challenging. Determining a clinically applicable BMI threshold for neonatal outcome risk assessment in fresh embryo transfer (fresh ET) cycles is meaningful; however, it remains undetermined. Therefore, this study aimed to identify an exploratory BMI threshold associated with increased risk of adverse neonatal outcomes after fresh ET, providing a preliminary reference for clinical risk communication.

**Methods:**

This retrospective cohort study included 2,195 women who underwent autologous fresh ET between June 2019 and December 2023. Multivariable regression analysis examined the association between prepregnancy BMI and neonatal outcomes. Smooth curve fitting and threshold effect analysis identified the exploratory BMI threshold. Adjusted analysis following propensity score matching (PSM) was conducted as a sensitivity analysis to validate the robustness of the results.

**Results:**

The multivariate regression analyses revealed that birthweight (adjusted β: 22.63, 95% CI: 14.53 to 30.73; P<0.001) and Z-score (adjusted β: 0.06, 95% CI: 0.04 to 0.08; P<0.001) were positively associated with increasing maternal BMI. Compared with the reference group (BMI 18.5-24.9 kg/m²), the incidence of macrosomia was increased by 2.25-fold in the BMI 25-29.9 kg/m² group (adjusted OR: 2.25, 95% CI: 1.29 to 3.93; P = 0.004) and by 4.56-fold in the BMI ≥30 kg/m² group (adjusted OR: 4.56, 95% CI: 2.26 to 9.22; P<0.001). Smooth curve fitting and threshold effect analysis revealed a significant increase in the odds of macrosomia when BMI exceeded 26.22 kg/m² (adjusted OR: 4.05, 95% CI: 2.47 to 7.29; P = 0.0009). PSM analysis confirmed that patients with a BMI >26.22 kg/m² (adjusted OR: 3.01, 95% CI: 2.02 to 4.47; P<0.001) had significantly higher odds of macrosomia compared to those with BMI ≤26.22 kg/m².

**Conclusion:**

Our findings suggest that a prepregnancy BMI exceeding approximately 26 kg/m² may be a potential predictor for an increased risk of macrosomia in singleton pregnancies conceived via fresh ET. This exploratory threshold may serve as a preliminary reference for risk communication and weight management counseling in women undergoing IVF treatment, though external validation is needed.

## Introduction

In recent decades, overweight and obesity have emerged as a critical global public health issue due to lifestyle and dietary changes, which are closely associated with metabolic and cardiovascular complications as well as reproductive health disorders such as menstrual irregularities, obstetric complications, and female infertility (1, 2). With the rising prevalence of obesity and growing evidence linking it to ovulation disorders and subfertility (3), an increasing proportion of obese patients are turning to assisted reproductive technologies (ART), particularly *in vitro* fertilization (IVF) (4). However, most analyses have confirmed that women with elevated body mass index (BMI) have significantly lower live birth rates and higher miscarriage rates compared to those with normal BMI following IVF treatment (5–7).

In addition to pregnancy outcomes, a growing number of studies are focusing on the correlation between prepregnancy BMI and neonatal outcomes in IVF. A recent multicentered cohort study, involving a large sample size of 115,287 cycles of both fresh and all subsequent frozen-thawed embryo transfers (FET), demonstrated that elevated prepregnancy BMI was an independent risk factor for fetal macrosomia, whereas the precise BMI threshold associated with increased macrosomia was not reported (8). Given that the hyper-estrogenic environment during ovarian stimulation in obese women may exacerbate metabolic dysfunction and that fresh embryo transfers may induce poorer embryo-endometrial synchrony compared to natural conception, establishing IVF-specific BMI cutoffs is of critical clinical importance. In Asian populations, a retrospective analysis of 16,240 singletons showed that a prepregnancy BMI ≥23 kg/m^2^ was significantly correlated with not only an increased incidence of macrosomia but also increased risks of preterm birth and large for gestational age (LGA) infants (9), which was defined as birth weight >90th percentile for gestational age and was clinically significant due to its associations with adverse outcomes such as shoulder dystocia, neonatal hypoglycemia, and childhood obesity. Similarly, Mackeen et al. reported that obesity significantly elevated the risk of macrosomia, hypertensive disorders of pregnancy (HDP), gestational diabetes mellitus (GDM), and composite adverse neonatal outcomes compared with normal-weight individuals (10). However, they proposed that the BMI threshold for increasing maternal and fetal risks was ≥40.0kg/m^2^ (10). In terms of birth defects, Chen et al. indicated that compared to those with prepregnancy BMI <25 kg/m², parental prepregnancy BMI ≥25 kg/m² was not only associated with an increased incidence of LGA in IVF/ICSI singleton pregnancies, but also elevated the risk of neonatal birth defects, particularly those in the musculoskeletal system (11). Recent studies have indicated that being underweight can also have adverse effects on neonatal outcomes following IVF treatment, such as increasing the likelihood of preterm births and the incidence of low birth weight (LBW) infants, with a BMI threshold considered to be less than 18.5 kg/m² (12–14).

From the aforementioned research, it can be observed that most current studies indicate that overweight status leads to an increase in large-size and high-birthweight newborns following IVF treatment, whereas underweight status results in an increase in small newborns. However, despite these findings, there may be variations in the BMI thresholds that influence neonatal outcomes after IVF. Some studies suggest that maintaining a BMI below 25 kg/m² is beneficial, while others propose thresholds of 23 kg/m², 30 kg/m² or 40 kg/m² (9–15). In these studies, populations are generally categorized by BMI based on World Health Organization (WHO) criteria, Asian standards, or physicians’ experiences. Beyond the conventional categorical groupings, establishing an evidence-based BMI cutoff value is important: it would enable clinicians to deliver individualized, actionable, and unequivocal recommendations to patients, thereby empowering more effective weight management strategies. While the current literature lacks a clinically actionable BMI cutoff value, to effectively guide pre-pregnancy counseling and weight management interventions. To address this gap, our study aims to determine the prepregnancy BMI cutoff value associated with neonatal outcomes in a Chinese population using adjusted smooth curve fitting and threshold effect analysis. Our ultimate goal is to provide clinicians and patients with an exploratory BMI threshold that facilitates targeted weight management strategies and enhances both clinical decision-making and neonatal health.

## Methods

### Study population

**Figure 1:**
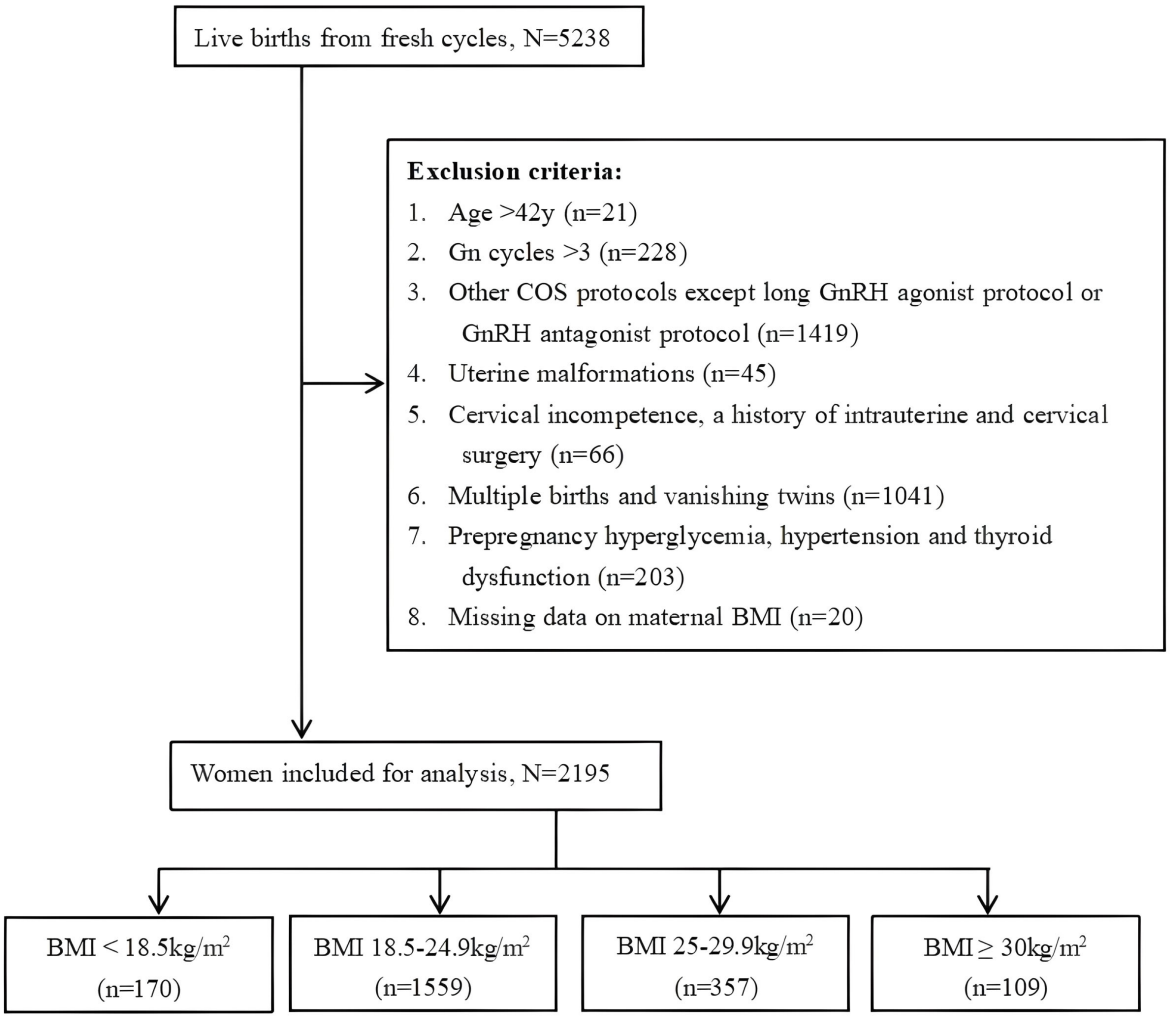
Flowchart of patient inclusion. Gn, gonadotropin; COS, controlled ovarian stimulation; GnRH-a, GnRH agonist; GnRH-A, GnRH antagonist; BMI, body mass index.

A retrospective cohort analysis was conducted at the Center of Assisted Reproduction at Tangdu Hospital of Fourth Military Medical University in China, covering the period from June 1, 2019, to December 31, 2023. Our study encompassed participants who satisfied the following criteria: autologous IVF/ICSI cycles, utilizing either the long luteal gonadotropin-releasing hormone agonist (GnRH-a) protocol or the GnRH antagonist protocol during the controlled ovarian stimulation (COS) process, being aged 42 years or younger at the time of oocyte retrieval, and having a live singleton birth following fresh ET. All participants were in the process of their initial three oocyte retrieval cycles. In this analysis, women with a prior history of prepregnancy hyperglycemia, hypertension and thyroid dysfunction were excluded to ensure that neonatal outcomes were not influenced by these factors. Furthermore, the study also excluded cases involving multiple births, vanishing twins, uterine malformations, cervical incompetence, a history of intrauterine or cervical surgery, and any cases where data on maternal BMI was missing. **Figure 1** presents a flowchart illustrating the patient selection process. Ultimately, 2195 participants were enrolled in this study.

### Treatment procedures

All patients underwent ovarian stimulation using either the long GnRH-a protocol or the GnRH antagonist protocol. The choice of the COS protocol was based on patient’s characteristics and physician’s experience. The initial Gn dosage was determined according to the patient’s sex hormone levels and transvaginal ultrasound results on the day of initiation. Both recombinant Gn and urinary exogenous Gn were utilized in the COS treatment. The daily dosage of Gn was adjusted according to the follicular development, as monitored by successive transvaginal ultrasound examinations, and serum hormone levels, including estradiol (E2), progesterone (P), and luteinizing hormone (LH). Continuous assessment of follicular sizes and hormone levels was conducted until the day of human chorionic gonadotropin (hCG) administration to trigger ovulation. Once at least three follicles with a diameter of ≥17 mm or two follicles with a diameter of ≥18 mm were observed via transvaginal ultrasound, all patients underwent ovulation triggering using either 250 micrograms (μg) of recombinant hCG (rhCG) or 5000–10000 international units (IU) of urinary hCG. Patients with peak E2 levels ≥6,000 pg/ml who received GnRH agonist-only ovulation triggering were routinely excluded from the study, as fresh ET was typically cancelled in these cases. Oocyte retrieval, guided by transvaginal ultrasound, was performed 35–37 hours after hCG administration. Fertilization assessment of the oocytes occurred approximately 12 to 17 hours after insemination or intracytoplasmic sperm injection (ICSI). Cleavage-stage embryos were transferred on the third day after oocyte retrieval, while blastocyst transfers generally took place on the fifth day (16). During the entire duration of the study, there were no notable alterations in the clinical and laboratory conditions, the culture media used for embryo culture, or the fresh ET techniques.

### Outcome measures and definitions

The baseline characteristics of the patients were collected, including the ages of the couple, maternal BMI, antimüllerian hormone (AMH) levels, type of infertility, duration and cause of infertility, and previous oocyte retrieval cycles. Data on COS cycle parameters encompassed the COS protocol, total Gn dosage, duration of stimulation, E2 and P levels on hCG trigger day, number of oocytes retrieved, developmental stages and numbers of embryos transferred, as well as the thickness and type of the endometrium.

Data on neonatal height, weight, sex, gestational age (GA), mode of delivery and pregnancy complication was collected for all singleton live births. GA was determined starting from the day of embryo transfer; for cleavage-stage embryo transfer, this is designated as day 17, and for blastocyst transfer, it is day 19 (17). Preterm delivery (PTD) and very PTD were defined as GA between 32–36 weeks and less than 32 weeks, respectively. Low birth weight (LBW), very LBW, and macrosomia were determined as birth weights below 2500 grams (g), below 1500 g, and 4000 g or above, respectively. Small for gestational age (SGA) and very SGA were defined as birth weights below the 10th and 3rd percentiles. Large for gestational age (LGA) and very LGA were defined as birth weights exceeding the 90th and 97th percentiles, respectively. Additionally, Z-scores were introduced to adjust birth weight for GA and neonatal sex, calculated using the following equation: Z-score = (x - μ)/σ, where x is the neonate’s weight, and μ and σ are the mean birth weight and standard deviation of infants of the same sex and GA, respectively. The calculation of birth weight percentiles and Z-scores refers to data from singleton births in China, stratified by GA and neonatal sex (18). Complications of pregnancy included hypertensive disorders complicating pregnancy, gestational diabetes, and intrahepatic cholestasis of pregnancy.

### Statistical analysis

Continuous variables are described as the means ± standard deviations (SDs) or medians and interquartile ranges (IQRs). Categorical data are presented as numbers and percentages. Differences among groups were compared via one-way analysis of variance (ANOVA) for continuous data and chi-square tests for categorical variables.

We selected the confounders on the basis of their associations with the outcomes of interest or a change in the effect estimate of more than 10%. Indicators related to birthweight, including fetal macrosomia, LBW, very LBW, SGA, very SGA, LGA and very LGA were adjusted for prior gonadotropin cycle, COS protocols, type of infertility, infertility duration, dosage of gonadotropins, E2 and P level on hCG day, number of oocytes retrieved, endometrial thickness, endometrial type, stage of embryos transferred, pregnancy complication, newborn sex and gestational age. Indicators related to GA, including preterm and very preterm were adjusted for maternal age, paternal age, type of infertility, infertility duration, infertility cause, prior gonadotropin cycle, COS protocols, P level on hCG day, number of oocytes retrieved, stage of embryos transferred, endometrial thickness, pregnancy complication and newborn sex.

Multiple linear regression analysis was conducted to explore the impact of maternal BMI (kg/m^2^) on continuous outcome indicators, including birthweight (g), GA (weeks), and Z-score. Logistic regression analysis was used to assess the categorical outcomes, such as LBW, very LBW, fetal macrosomia, preterm, very preterm, SGA, very SGA, LGA, and very LGA, while accounting for potential confounding variables. Smooth curve fitting and threshold effect analysis were performed to evaluate the potential threshold of BMI that significantly affected the neonatal outcomes in fresh ET cycles. The inflection point was identified using a two-piecewise logistic regression model. The algorithm systematically searched for the best-fitting breakpoint by evaluating candidate BMI values across the distribution and selected the one that maximized the model likelihood.

In order to avoid the interference of confounders as much as possible, we introduced the adjustment analysis after propensity score matching (PSM) as a sensitivity analysis to test the robustness of the primary findings. Matching was performed between the two groups with the use of 1:1 matching protocol and width equal to 0.05 of the standard deviation of the logit of the propensity score. The propensity score was estimated using a logistic regression model that included the following baseline covariates: maternal age, paternal age, type and duration of infertility, prior gonadotropin cycle, COS protocols dosage of gonadotropins, E2 and P level on hCG day, number of oocytes retrieved, stage of embryos transferred, endometrial thickness and newborn sex.

All statistical analyses were conducted utilizing EmpowerStats (available at www.empowerstats.com, X&Y solutions, Inc., Boston MA) and R software, version 3.6.1 (accessible at http://www.r-project.org). Statistical significance was determined at a P value of <0.05.

## Results

In this study, a total of 2,195 fresh IVF cycles were included and stratified into four groups in terms of women’s BMI (kg/m^2^) according to the WHO criteria. 170 live-born singletons were categorized as Group 1 (BMI < 18.5), 1559 as Group 2 (BMI 18.5-24.9), 357 as Group 3 (BMI 25-29.9), and 109 as Group 4 (BMI ≥30), respectively.

The baseline characteristics of the patients, parameters of the COS cycles, and neonatal outcomes are shown in **Table 1**. When compared to Group 1, women in the higher BMI groups were older and had experienced infertility for a longer duration. With respect to cycle parameters, the groups with higher BMI exhibited higher total Gn dosage, a longer duration of stimulation, lower E2 levels on the day of hCG administration, a greater number of cleavage embryos transferred, and a thicker endometrium. In relation to neonatal outcomes, as BMI increased, there was a gradual rise in birthweight (g), Z-score, and the incidence of macrosomia, LGA, cesarean sections and pregnancy complications, while the proportion of SGA decreased. All these differences were statistically significant (P<0.05).

**Table 1:** Patient baseline characteristics, COS cycle parameters and neonatal outcomes by different groups of prepregnancy BMI (n=2195).

Characteristics	< 18.5kg/m2	18.5-24.9kg/m2	25-29.9kg/m2	≥30kg/m2	P value
Baseline characteristics					
Maternal age (y)	30.25 ± 3.58	31.55 ± 3.91	31.75 ± 3.95	31.41 ± 4.04	<0.001
Paternal age (y)	31.48 ± 4.02	32.97 ± 4.58	32.81 ± 4.37	33.98 ± 5.14	<0.001
Maternal BMI (kg/m2)	17.71 ± 0.68	21.76 ± 1.70	26.63 ± 1.16	31.56 ± 1.50	<0.001
AMH (ng/ml)	3.13 ± 2.29	2.99 ± 2.16	3.19 ± 2.30	2.67 ± 2.19	0.249
Type of infertility					0.020
Primary	99 (58.24%)	737 (47.30%)	168 (47.19%)	65 (59.63%)	
Secondary	71 (41.76%)	821 (52.70%)	188 (52.81%)	44 (40.37%)	
Infertility duration (y)	3.19 ± 2.60	3.45 ± 2.73	3.87 ± 3.08	4.45 ± 3.48	0.003
Infertility cause					0.152
Female	83 (48.82%)	897 (57.54%)	188 (52.66%)	67 (61.47%)	
Male	38 (22.35%)	359 (23.03%)	88 (24.65%)	20 (18.35%)	
Mixed	37 (21.76%)	208 (13.34%)	53 (14.85%)	16 (14.68%)	
Unexplained	12 (7.06%)	95 (6.09%)	28 (7.84%)	6 (5.50%)	
Prior gonadotropin cycle	1.19 ± 0.47	1.20 ± 0.52	1.18 ± 0.46	1.26 ± 0.57	0.742
COS cycle parameters					
COS protocols					0.269
GnRH-a long protocol	108 (63.53%)	991 (63.57%)	213 (59.66%)	53 (48.98%)	
Antagonist protocol	60 (35.29%)	523 (33.55%)	131 (36.69%)	56 (51.02%)	
Dosage of gonadotropins (IU)	1659.06 ± 915.84	2022.57 ± 1026.36	2348.32 ± 1001.23	3068.70 ± 1324.99	<0.001
Stimulation duration (days)	11.22 ± 2.03	11.60 ± 2.04	12.01 ± 2.13	12.42 ± 2.20	<0.001
E2 level on hCG day (pg/ml)	2789.75 ± 1124.03	2571.09 ± 1125.39	2442.85 ± 1121.46	2270.05 ± 1506.83	0.003
P level on hCG day (ng/ml)	0.68 ± 0.33	0.65 ± 0.45	0.63 ± 0.29	0.49 ± 0.27	0.179
Number of oocytes retrieved	9.70 ± 3.47	9.61 ± 3.61	10.13 ± 3.72	9.47 ± 3.57	0.098
Retrieved MII Oocytes	8.45 ± 3.18	8.44 ± 3.32	8.77 ± 3.37	8.14 ± 3.30	0.335
Fertilization method					0.958
IVF	111 (65.29%)	1014 (65.04%)	225 (63.03%)	73 (66.97%)	
ICSI	52 (30.59%)	457 (29.31%)	109 (30.53%)	31 (28.44%)	
IVF+ICSI	7 (4.12%)	88 (5.64%)	23 (6.44%)	5 (4.59%)	
Stage of embryos transferred					0.009
D3	84 (49.41%)	732 (46.95%)	151 (42.30%)	73 (66.97%)	
D4	17 (10.00%)	149 (9.56%)	51 (14.29%)	9 (8.26%)	
D5	69 (40.59%)	678 (43.49%)	155 (43.42%)	27 (24.77%)	
Number of embryos transferred					0.116
1	86 (50.59%)	791 (50.74%)	199 (55.74%)	36 (33.03%)	
2	84 (49.41%)	767 (49.20%)	158 (44.26%)	73 (66.97%)	
3	0 (0.00%)	1 (0.06%)	0 (0.00%)	0 (0.00%)	
Endometrial thickness (mm)	10.12 ± 1.90	10.31 ± 1.80	10.54 ± 1.83	10.71 ± 2.31	0.031
Endometrial type					<0.001
A	8 (4.73%)	31 (2.04%)	5 (1.43%)	0 (0.00%)	
A-B	8 (4.73%)	57 (3.75%)	11 (3.15%)	2 (1.83%)	
B	70 (41.42%)	542 (35.66%)	105 (30.09%)	22 (20.18%)	
B-C	73 (43.20%)	761 (50.07%)	178 (51.00%)	65 (59.63%)	
C	10 (5.92%)	129 (8.48%)	50 (14.33%)	20 (18.35%)	
Neonatal outcomes indicators					
Newborn sex					0.939
Male	93 (54.71%)	833 (53.43%)	189 (52.94%)	62 (56.88%)	
Female	77 (45.29%)	726 (46.57%)	168 (47.06%)	47 (43.12%)	
Newborn height (cm)	49.89 ± 1.64	50.05 ± 2.33	50.19 ± 2.64	49.65 ± 3.39	0.348
GA (weeks)	39.09 ± 1.42	39.00 ± 1.69	38.91 ± 1.80	38.48 ± 1.99	0.119
Birthweight (g)	3178.35 ± 406.00	3276.38 ± 494.73	3344.48 ± 565.65	3298.98 ± 693.64	0.005
Z-score	-0.10 ± 0.90	0.19 ± 1.04	0.40 ± 1.15	0.41 ± 1.44	<0.001
Very low birthweight (<1500g)	0 (0.00%)	10 (0.64%)	3 (0.84%)	2 (1.83%)	0.431
Low birthweight (<2500g)	8 (4.71%)	59 (3.78%)	15 (4.20%)	7 (6.42%)	0.795
Fetal macrosomia (≥4000g)	4 (2.35%)	100 (6.41%)	43 (12.04%)	13 (11.93%)	<0.001
Very preterm(<32w)	0 (0.00%)	12 (0.78%)	5 (1.41%)	2 (1.83%)	0.296
Preterm(32-36w)	16 (9.52%)	102 (6.60%)	25 (7.06%)	11 (10.09%)	0.307
Small for gestational age	15 (8.93%)	71 (4.60%)	12 (3.39%)	2 (1.83%)	0.030
Very small for gestational age	2 (1.19%)	28 (1.81%)	8 (2.26%)	4 (3.67%)	0.567
Large for gestational age	2 (1.19%)	97 (6.28%)	40 (11.20%)	12 (11.01%)	<0.001
Very large for gestational age	3 (1.79%)	57 (3.69%)	20 (5.65%)	9 (8.26%)	0.063
Mode of delivery					<0.001
Vaginal	85 (50.00%)	549 (35.26%)	103 (28.85%)	18 (16.51%)	
Caesarean section	85 (50.00%)	1008 (64.74%)	254 (71.15%)	91 (83.49%)	
GDM	8 (4.7%)	107 (6.86%)	42 (11.76%)	17 (15.60%)	<0.001

GDM, Gestational Diabetes Mellitus.

The univariate linear analyses summarized in **Supplementary Table 1** indicated that there were eleven factors significantly influenced birthweight, including maternal BMI, type of infertility, infertility duration, COS protocols, E2 and P level on hCG day, number of oocytes retrieved, stage of embryos transferred, endometrial thickness, newborn sex, and gestational age. Additionally, ten factors were found to significantly affect gestational age, including couple’s age, maternal BMI, type of infertility, infertility duration, COS protocols, P level on hCG day, number of oocytes retrieved, endometrial thickness, and newborn sex.

The multivariate regression analysis results for neonatal outcomes are presented in **Table 2**. The birthweight demonstrated a positive correlation with the rising maternal BMI, even after considering confounding factors (adjusted β: 22.63, 95% CI: 14.53 to 30.73; P < 0.001). Furthermore, when BMI was treated as a categorical variable, birthweight still exhibited an increasing trend in the higher BMI groups (3212.39g vs. 3268.79g vs. 3346.56g vs. 3495.02g, P trend=0.006). Specifically, significant increases in birthweight were observed in the BMI categories of 25-29.9 kg/m² (adjusted β: 78.88, 95% CI: 13.76 to 144.00; P = 0.0177) and ≥ 30 kg/m² (adjusted β: 231.50, 95% CI: 63.33 to 399.68; P = 0.0071), compared to the normal BMI range of 18.5-24.9 kg/m^2^. The trend of increasing Z-scores in positive correlation with maternal BMI was similar to that observed for birthweight. For every 1 kg/m^2^ increased in BMI, the Z-score increased by 0.06 (adjusted β: 0.06, 95% CI: 0.04 to 0.08; P<0.001). Additionally, a notable increase in the incidence of fetal macrosomia was also observed with higher BMI values (adjusted OR: 1.35, 95% CI: 1.08 to 1.64; P = 0.0002). When BMI was stratified into four groups, compared to the reference group (18.5-24.9 kg/m^2^), the odds of macrosomia significantly increased in the group with BMI ranging from 25-29.9 kg/m^2^ (adjusted OR: 2.25, 95% CI: 1.29 to 3.93; P = 0.004) and even more so in the group with BMI ≥ 30 kg/m^2^ (adjusted OR: 4.56, 95% CI: 2.26 to 9.22; P<0.0001). The results of multiple regression analysis also indicated that the GA decreased and the rates of LGA increased with the rising BMI. However, due to the potentially modest range of variation in the two variables, no statistically significant differences were observed among the groups when BMI was categorized into four levels.

**Table 2:** Multivariable regression analysis for neonatal outcomes by prepregnancy BMI(n=2195).

Characteristics	Non-adjusted β / OR (95% CI)	P value	Adjusted β / OR (95% CI)	P value
Birthweight (g)	15.99 (9.00, 22.99)	<0.0001	22.63 (14.53, 30.73)	<0.0001
18.5-24.9 kg/m2	Ref		Ref	
< 18.5 kg/m2	-98.03 (-178.18, -17.89)	0.0166	-48.99 (-138.44, 40.46)	0.2833
25-29.9 kg/m2	68.10 (9.88, 126.32)	0.0220	78.88 (13.76, 144.00)	0.0177
≥30 kg/m2	22.59 (-121.37, 166.56)	0.7584	231.50 (63.33, 399.68)	0.0071
Z-score	0.05 (0.03, 0.06)	<0.0001	0.06 (0.04, 0.08)	<0.0001
18.5-24.9 kg/m2	Ref		Ref	
< 18.5 kg/m2	-0.29 (-0.46, -0.12)	0.0009	-0.14 (-0.37, 0.09)	0.2260
25-29.9 kg/m2	0.21 (0.09, 0.34)	0.0007	0.24 (0.08, 0.41)	0.0042
≥30 kg/m2	0.23 (-0.07, 0.53)	0.1390	0.63 (0.21, 1.06)	0.0037
Gestational age (w)	-0.03 (-0.05, -0.00)	0.0372	-0.04 (-0.07, -0.01)	0.0233
18.5-24.9 kg/m2	Ref		Ref	
< 18.5 kg/m2	0.08 (-0.19, 0.35)	0.5491	0.17 (-0.20, 0.54)	0.3803
25-29.9 kg/m2	-0.10 (-0.29, 0.10)	0.3398	-0.12 (-0.39, 0.14)	0.3581
≥30 kg/m2	-0.53 (-1.01, -0.04)	0.0331	-0.67 (-1.36, 0.02)	0.0555
Fetal macrosomia (≥4000g)	1.13 (1.07, 1.18)	<0.0001	1.35 (1.08, 1.64)	0.0002
18.5-24.9 kg/m2	Ref		Ref	
< 18.5 kg/m2	0.35 (0.13, 0.97)	0.0429	0.47 (0.14, 1.57)	0.2180
25-29.9 kg/m2	2.00 (1.37, 2.91)	0.0003	2.25 (1.29, 3.93)	0.0044
≥30 kg/m2	1.66 (0.64, 4.27)	0.2953	4.56 (2.26, 9.22)	<0.0001
LGA (>90th percentile)	1.02 (0.91, 1.10)	0.0566	1.05 (1.00, 1.21)	0.0431
18.5-24.9 kg/m2	Ref		Ref	
< 18.5 kg/m2	0.18 (0.04, 0.74)	0.0170	0.15 (0.02, 1.09)	0.0603
25-29.9 kg/m2	2.06 (1.41, 3.02)	0.0002	1.54 (0.89, 2.66)	0.1256
≥30 kg/m2	1.70 (0.66, 4.38)	0.2743	2.85 (0.86, 9.42)	0.0868
Very LGA (>97th percentile)	1.56 (0.93, 2.64)	0.0641	1.69 (0.91, 4.53)	0.0980
SGA (<10th percentile)	0.92 (0.85, 0.98)	0.0161	0.93 (0.84, 1.02)	0.1385
Very SGA (<3rd percentile)	1.04 (0.94, 1.15)	0.4189	1.01 (0.86, 1.18)	0.9238
LBW (<2500g)	0.99 (0.93, 1.07)	0.8658	0.92 (0.80, 1.04)	0.1861
Very LBW (<1500g)	1.18 (1.02, 1.36)	0.0250	1.32 (0.25, 7.00)	0.7433
Preterm (32-36w)	0.99 (0.94, 1.05)	0.8511	1.02 (0.94, 1.10)	0.6844
Very preterm (<32w)	1.15 (1.01, 1.32)	0.0302	1.83 (0.64, 5.22)	0.2597
Newborn height	0.02 (-0.02, 0.05)	0.3110	0.01 (-0.04, 0.05)	0.7849
Newborn sex	0.99 (0.97, 1.02)	0.6724	1.00 (0.96, 1.04)	0.8776

Multiple linear regression analysis was conducted to explore the impact of maternal BMI (kg/m2) on continuous outcome indicators. Logistic regression analysis was used to assess the categorical outcomes.

Analyses of birthweight, fetal macrosomia, LBW, very LBW, SGA, very SGA, LGA and very LGA were adjusted for prior gonadotropin cycle, COS protocols, type of infertility, infertility duration, dosage of gonadotropins, E2 and P level on hCG day, number of oocytes retrieved, endometrial thickness, endometrial type, stage of embryos transferred, pregnancy complication, newborn sex and gestational age.

Analyses of GA, preterm and very preterm were adjusted for maternal age, paternal age, type of infertility, infertility duration, infertility cause, prior gonadotropin cycle, COS protocols, P level on HCG day, number of oocytes retrieved, stage of embryos transferred, pregnancy complication, endometrial thickness and newborn sex.

OR, odds ratio; CI, confidence interval; Ref, reference group.

**Supplementary Table 2** showed the tests for trend across BMI categories for birthweight, Z-score, macrosomia, and LGA. After adjustment for confounders, birthweight, Z-score, and macrosomia showed significant increasing trends across BMI categories, whereas no significant trend was observed for LGA. We also performed sensitivity analyses with stepwise covariate adjustment in **Supplementary Table 3**. The results were consistent across all three models. Compared with the normal weight group (BMI 18.5–24.9 kg/m²), the group of BMI 25–29.9 kg/m² had significantly higher birthweight, Z-score, and macrosomia risk. The BMI ≥30 kg/m² group showed even larger effects. Effect estimates remained stable across models, demonstrating the robustness of our findings.

**Figure 2:**
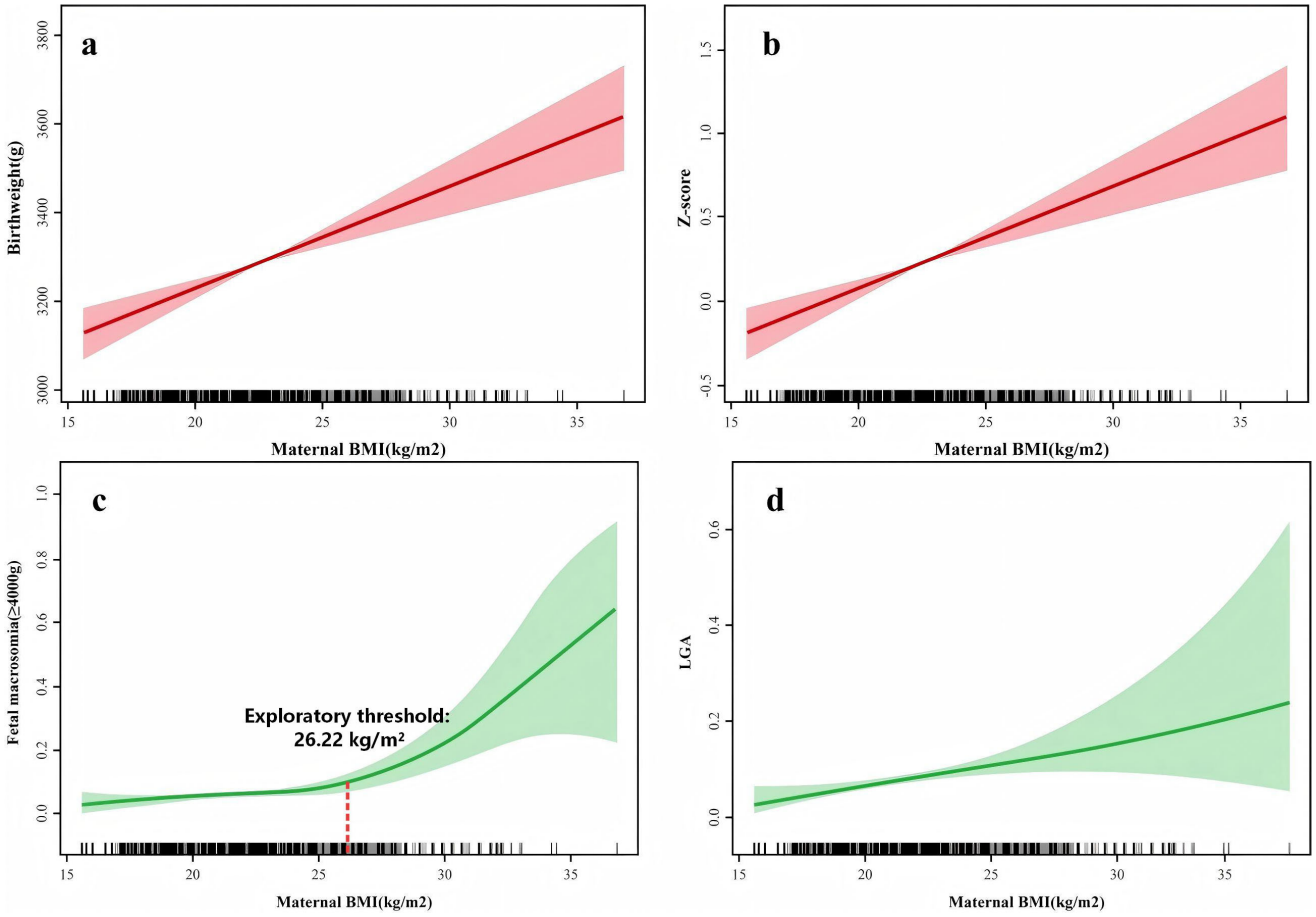
The relationship between prepregnancy maternal BMI(kg/m^2^) and adjusted birthweight and Z-score, the adjusted incidence of fetal macrosomia and LGA. Analyses were adjusted for prior gonadotropin cycle, type of infertility, infertility duration, COS protocols, dosage of gonadotropins, E2 and P level on hCG trigger day, number of oocytes retrieved, endometrial thickness, endometrial type, stage of embryos transferred, pregnancy complication, newborn sex and gestational age. **(a)** The relationship between prepregnancy maternal BMI and adjusted birthweight. **(b)** The relationship between prepregnancy maternal BMI and adjusted Z-score. **(c)** The relationship between prepregnancy maternal BMI and the adjusted incidence of fetal macrosomia. **(d)** The relationship between prepregnancy maternal BMI and the adjusted incidence of LGA. The vertical dashed line indicates the exploratory BMI threshold of approximately 26.22 kg/m²identified by the threshold effect analysis. The number of observations is relatively small in the BMI ranges: 357samples in 25-29.9 kg/m²and 109 samples in >30 kg/m², which explains the wide confidence intervals observed in these regions.

**Figure 2** showed the relationship between prepregnancy maternal BMI and adjusted birthweight (a) and Z-score (b), the adjusted incidence of fetal macrosomia (c) and LGA (d). It can be observed that there is a significant positive correlation between maternal BMI and both birthweight and Z-score. According to the adjusted incidence of fetal macrosomia, a curvilinear relationship existed between the two. The curve exhibited a gentle slope in the initial segment, followed by a sharp rise in the latter segment, indicating that the incidence of macrosomia remained relatively stable at lower BMI levels but increased markedly once BMI exceeds a certain cutoff value. The curve illustrated the relationship between prepregnancy BMI and the adjusted rates of LGA. It can be seen that the overall trend of this curve appeared more gradual compared to the curve of the incidence of macrosomia, indicating that the increase in LGA incidence with rising BMI was less steep than that of macrosomia. This findings were consistent with the multivariate analysis results presented in **Table 2**.

When combined with the threshold effect identified through piece-wise logistic regression, as shown in **Table 3**, it displayed that when BMI exceeded 26.22 kg/m², the incidence of macrosomia increased significantly with further increases in BMI, with each 1 kg/m² increase associated with a 4.05-fold increase in the odds of macrosomia (OR: 4.05, 95% CI: 2.47–7.29, p = 0.0009). In contrast, when BMI was below 26.22 kg/m², the incidence of macrosomia remained at a relatively stable and low level, with no significant association between increasing BMI and the risk of macrosomia. This suggested that approximately 26 kg/m^2^ may serve as a potential cutoff value where BMI began to significantly impact the incidence of fetal macrosomia. However, this pattern was not observed for LGA. At higher BMI values, the confidence intervals widen substantially due to the relatively smaller number of observations in this range, indicating limited precision of the estimates. Therefore, findings in the upper BMI range should be interpreted with caution.

**Table 3:** Threshold effect of prepregnancy BMI on fetal macrosomia and LGA using piece-wise logistic regression.

Characteristics	Non-adjusted OR (95% CI)	P value	Adjusted OR (95% CI)	P value
Fetal macrosomia				
BMI ≤ 26.22 kg/m2	1.54 (0.91, 3.07)	0.0743	1.69 (0.59, 3.19)	0.1697
BMI > 26.22 kg/m2	2.91 (0.84, 5.29)	0.3461	4.05 (2.47, 7.29)	0.0009
LGA				
BMI ≤ 26.22 kg/m2	1.02 (0.79, 1.35)	0.5673	1.47 (0.83, 1.89)	0.2478
BMI > 26.22 kg/m2	0.99 (0.80, 1.23)	0.9448	1.32 (0.97, 1.78)	0.0781

Analyses of fetal macrosomia and LGA were adjusted for prior gonadotropin cycle, COS protocols, type of infertility, infertility duration, dosage of gonadotropins, E2 and P level on HCG day, number of oocytes retrieved, endometrial thickness, endometrial type, stage of embryos transferred, pregnancy complication, newborn sex and gestational age.

OR, odds ratio; CI, confidence interval.

The introduction of PSM aimed to identify patient cohorts with similar baseline characteristics, including indicators significantly associated with BMI as shown in **Supplementary Table 4**. Based on the results in **Table 3**, the data were divided into two groups: one with BMI ≤ 26.22 kg/m^2^, and the other with BMI > 26.22 kg/m^2^. Before matching, the BMI > 26.22 kg/m² group included 359 patients, and the BMI ≤ 26.22 kg/m² group included 1,836 patients. After matching, 226 patients in each group were successfully matched. 133 patients in the BMI > 26.22 kg/m² group and 1,610 patients in the BMI ≤ 26.22 kg/m² group were unmatched and excluded. After PSM, most covariates achieved good balance (SMD < 0.1). However, small to moderate imbalances remained for three variables, including infertility duration (SMD = 0.202), dose of Gn (SMD = 0.235), and endometrial thickness (SMD = 0.179). These residual imbalances were addressed by including these variables as covariates in the subsequent regression analyses. The regression analysis results after PSM, as presented in **Table 4**, indicated that after adjusting for baseline data and propensity scores, when BMI > 26.22 kg/m^2^, there were significant increases in birthweight (adjusted β: 107.52, 95% CI: 22.18 to 192.86; P = 0.0139), Z-score (adjusted β: 0.32, 95% CI: 0.10 to 0.54; P = 0.0044), and the incidence of macrosomia (adjusted OR: 3.01, 95% CI: 2.02 to 4.47; P < 0.0001) compared to those with BMI ≤ 26.22 kg/m^2^. These findings were consistent with the results presented in **Table 2** and **Table 3**.

**Table 4:** Association between prepregnancy BMI and neonatal outcomes after Propensity Score Matching (PSM).

Characteristics	Effect Estimate, β / OR (95% CI)					
	Non-adjusted	P value	Baseline characteristics and COS cycle parameters Adjusted	P value	Propensity score adjusted	P value
Birthweight (g)						
BMI ≤ 26.22 kg/m2	Ref		Ref		Ref	
BMI > 26.22 kg/m2	77.69 (-21.39, 176.78)	0.1250	101.79 (16.88, 186.69)	0.0193	107.52 (22.18, 192.86)	0.0139
Z-score						
BMI ≤ 26.22 kg/m2	Ref		Ref		Ref	
BMI > 26.22 kg/m2	0.29 (0.08, 0.50)	0.0070	0.31 (0.09, 0.52)	0.0058	0.32 (0.10, 0.54)	0.0044
Fetal macrosomia (≥4000g)						
BMI ≤ 26.22 kg/m2	Ref		Ref		Ref	
BMI > 26.22 kg/m2	1.91 (0.87, 4.19)	0.1051	2.90 (1.72, 4.17)	0.0069	3.01 (2.02, 4.47)	<0.0001
Gestational age (w)						
BMI ≤ 26.22 kg/m2	Ref		Ref		Ref	
BMI > 26.22 kg/m2	-0.25 (-0.56, 0.06)	0.1151	-0.28 (-0.61, 0.05)	0.0965	-0.29 (-0.62, 0.04)	0.0895
LBW (<2500g)						
BMI ≤ 26.22 kg/m2	Ref		Ref		Ref	
BMI > 26.22 kg/m2	1.15 (0.41, 3.22)	0.7930	0.44 (0.08, 2.41)	0.3452	0.47 (0.09, 2.52)	0.3804
Preterm (32-36w)						
BMI ≤ 26.22 kg/m2	Ref		Ref		Ref	
BMI > 26.22 kg/m2	1.33 (0.63, 2.81)	0.4510	1.44 (0.63, 3.30)	0.3864	1.46 (0.63, 3.37)	0.3720
SGA(<10^th^ percentile)						
BMI ≤ 26.22 kg/m2	Ref		Ref		Ref	
BMI > 26.22 kg/m2	0.72 (0.28, 1.83)	0.4898	0.69 (0.24, 2.05)	0.5083	0.67 (0.22, 1.99)	0.4683
Very SGA(<3^rd^ percentile)						
BMI ≤ 26.22 kg/m2	Ref		Ref		Ref	
BMI > 26.22 kg/m2	0.60 (0.14, 2.53)	0.4841	0.04 (0.05, 1.06)	0.243	0.03 (0.04, 1.03)	0.1090
LGA(>90^th^ percentile)						
BMI ≤ 26.22 kg/m2	Ref		Ref		Ref	
BMI > 26.22 kg/m2	2.05 (1.02, 4.12)	0.0438	2.01 (0.91, 4.44)	0.0849	2.01 (0.91, 4.47)	0.0856
Very LGA(>97^th^ percentile)						
BMI ≤ 26.22 kg/m2	Ref		Ref		Ref	
BMI > 26.22 kg/m2	1.50 (0.68, 3.30)	0.3178	1.38 (0.55, 3.45)	0.4893	1.40 (0.56, 3.54)	0.4714

The matching variables include maternal age, paternal age, infertility duration, type of infertility, prior gonadotropin cycle, COS protocols, dosage of gonadotropins, E2 and P level on hCG trigger day, number of oocytes retrieved, stage of embryos transferred, endometrial thickness, pregnancy complication, and newborn sex.

OR, odds ratio; CI, confidence interval; Ref, reference group.

## Discussion

The primary objective of this study, which included 2,195 singleton births from infertile women under 42 years of age, was to explore the prepregnancy BMI threshold associated with neonatal outcomes following fresh ET in a Chinese population. Our results revealed a significant positive correlation between maternal BMI and the incidence of macrosomia, particularly when BMI exceeded 26.22 kg/m^2^. These findings suggest that a prepregnancy BMI exceeding approximately 26 kg/m² may be a potential predictor for an increased risk of macrosomia in singleton pregnancies conceived via fresh ET. This exploratory threshold may serve as a preliminary reference for risk communication and weight management counseling in women undergoing IVF treatment.

In recent years, the delay in childbearing and the growing prevalence of overweight and obesity have led to heightened focus on the link between a mother’s BMI and fetal safety, extending beyond mere pregnancy outcomes. A recent retrospective cohort study, which collected data from five major academic reproductive medicine centers in China, indicated that, maternal BMI had no association with neonatal outcomes except for fetal macrosomia (8), which was consistent with our findings. The research included both fresh and frozen embryo transfer cycles, while to minimize bias, our research exclusively involved fresh embryo transfer cycles. Furthermore, this study did not perform intergroup comparisons of neonatal outcomes stratified by BMI; consequently, the BMI cutoff value associated with an increased incidence of macrosomia was not reported (8). Yang et al. discovered that prepregnancy overweight and obesity according to Asian criteria with BMI≥ 23kg/m^2^ significantly elevated the risks of preterm birth, macrosomia, and LGA in frozen embryo transfer (FET) cycles (9). Similarly, Chen et al. reported that a parental prepregnancy BMI of ≥ 25 kg/m^2^ was associated with a higher incidence of LGA in IVF/ICSI singletons, specifically in fresh ET cycles (11). Our study also found this trend, showing an increasing incidence of LGA with a higher prepregnancy maternal BMI. However, after adjusting for confounding factors, both multivariate regression analysis and analysis following PSM showed no significant correlation between maternal BMI and LGA. This discrepancy in the association of the exploratory BMI threshold identified in our study with macrosomia versus LGA may stem from fundamental differences in their definitions: macrosomia is based on absolute birth weight (>4000 g), whereas LGA is adjusted for gestational age and sex. Elevated prepregnancy BMI may primarily drive absolute birthweight rather than relative growth adjusted for gestational age. Therefore, this BMI threshold may be outcome-specific and currently applies only to macrosomia. Future studies should further investigate the applicability of different BMI thresholds across different neonatal outcomes. Notably, all the aforementioned studies were based on data from China.

What about the situation in other countries? Data from the national ART Surveillance System in the United States revealed that that in fresh autologous cycles resulting in singleton births, both underweight (BMI<18.5 kg/m^2^) and obese women (BMI >30 kg/m^2^) had a higher risk of LBW and PTD (12). A large-scale study from a validated maternity database system in London, involving 678,811 participants, demonstrated that in obese women, defined as a BMI >25 kg/m^2^, the risks of LGA and intrauterine death increased significantly with the higher BMI (19). Studies on spontaneous pregnancies also indicated that prepregnancy obesity with BMI >30kg/m^2^ was associated with fetal macrosomia, LGA (20, 21), and PTD (22, 23). Evidently, regardless of whether the pregnancy occurred in Asia or Europe and America, and irrespective of whether it was achieved through IVF treatment, infants born to overweight or obese women before pregnancy were mostly larger in size and higher in birthweight. Our study, through various analytical methods, showed an increase in birthweight and the incidence of macrosomia, aligning with previous findings.

The definition of underweight is relatively consistent across all populations, defined as <18.5kg/m^2^. The safety of offspring born to mothers who were underweight before pregnancy has also received widespread attention. In spontaneous pregnancies, previous observational studies indicated that underweight status was a risk factor for PTD (24–26). Among women undergoing IVF, most research found that underweight before pregnancy still increased the risk of PTD and LBW in infants (12–14). Our study showed a slight increasing trend for PTD and LBW in underweight women, but these differences were not statistically significant. Additionally, we observed a significant increase in SGA infants among underweight patients. However, after adjusting for confounding factors, both multivariate regression analysis and analysis following PSM showed that underweight status did not increase the risk of SGA. This inconsistency may be attributed to differences in the study populations selected. Wu’s study focused on patients with endometriosis (13), while Fukui’s research comprised Japanese individuals with dietary and lifestyle habits that were substantially different from those of Chinese (14). Whereas, Romanski’s study included participants similar to ours, consisting of women undergoing autologous IVF with fresh ET (27). Their findings were also consistent with our results, showing no association between underweight and neonatal outcomes. Importantly, it should be still noted that the number of singleton deliveries reported in our underweight groups was relatively small, potentially lacking the statistical power necessary to detect subtle differences in neonatal outcomes.

Studies from different regions categorized participants into groups based on different BMI cutoff values for classification. Some studies adopted Asian standards for grouping (2, 8), others followed WHO criteria (12, 19), and some simply divided into two groups based on a maternal BMI threshold of 25 kg/m^2^ (11, 15). Regardless of the grouping method used, there is currently a lack of exploration into the dose-response relationship between the two variables and the precise cutoff values of BMI that impact neonatal outcomes. Our study is the first to use adjusted curve fitting and threshold effect analysis to determine the maternal BMI cutoff value at which the incidence of macrosomia significantly increases. Ultimately, we found that a maternal BMI exceeding approximately 26 kg/m² may be an independent predictor for a remarkable increase in the odds of macrosomic infants following fresh ET. The exploratory BMI threshold (approximately 26 kg/m²) falls within the WHO overweight range (25-29.9 kg/m²). Consistent with the WHO classification, this threshold also indicates that higher BMI is associated with increased risk of macrosomia. However, unlike the broad WHO category, this threshold further suggests that the risk is not uniformly distributed within the overweight range: the risk of macrosomia accelerates once BMI exceeds approximately 26 kg/m². Thus, this exploratory threshold may serve as a refinement of the WHO overweight classification, offering a more specific reference point for weight management counseling.

The underlying mechanisms by which higher maternal BMI affect neonatal outcomes are not yet fully understood. Previous studies reported that insulin resistance in obese women can lead to an increase in nutrients supplied to the fetus through the placenta (21). The available data showed that increased prepregnancy maternal insulin resistance, accompanied by hyperinsulinemia, inflammation, and oxidative stress, are thought to contribute to early placental and fetal dysfunction (28). Hexokinase is the first and one of the rate-limiting enzymes of glycolysis. Recent research revealed that placental hexokinase activity, being crucial for uteroplacental retention of glucose, correlated positively with prepregnancy maternal BMI and birthweight (29). This suggested that overweight or obese patients may increase the birthweight by increasing placental hexokinase activity. Much additional research is still needed to explore the mechanisms by which overweight and underweight status impact neonatal health outcomes.

The primary strength of this study is the first to explore the dose-response relationship between maternal BMI and neonatal outcomes, and to report an exploratory BMI threshold of approximately 26 kg/m^2^ that affects the odds of macrosomia significantly. Therefore, this threshold may provide a preliminary clinically meaningful reference for weight management counseling in infertile women undergoing fresh ET. A second strength of this study is that, to further enhance the reliability of our findings on the relationship between maternal BMI and neonatal outcomes, we employed not only multivariate regression analysis but also PSM analysis. The consistency between the two analytical methods indicates a strong positive correlation between maternal BMI and both birthweight and the incidence of macrosomia. However, several limitations of this study should be acknowledged. First, it is a single-center retrospective design. And we lacked data on gestational weight gain (GWG). GWG is a key factor influencing birthweight and the risk of macrosomia. Failure to include GWG in the analysis may have introduced residual confounding and could affect the accuracy of the estimated association between prepregnancy BMI and macrosomia. Therefore, readers should interpret our findings with caution in light of the absence of GWG data, and our results warrant validation in future studies that include detailed information on gestational weight management. Second, it should be noted that the proposed BMI threshold was internally derived from a single retrospective cohort and lacks external validation. Thus, this finding should be interpreted with caution until replicated in independent, preferably multicenter, cohorts. Third, it should be noted that the starting population of this study consisted of women who achieved singleton live births following fresh ET, rather than all women who underwent fresh ET. Excluded non-pregnant or pregnancy-failure women may differ in baseline characteristics from those included. So, our findings apply only to women who successfully achieved singleton live births and cannot be directly generalized to all women undergoing IVF or fresh ET. Furthermore, owing to limitations in the data collection protocol, secondary neonatal outcomes (e.g., shoulder dystocia, NICU admission) were inadequately documented and consequently excluded from analysis, potentially limiting the clinical applicability of our findings.

## Conclusions

In conclusion, this retrospective study suggests that in singleton infants conceived via fresh ET by women under 42 years of age, a maternal prepregnancy BMI exceeding approximately 26 kg/m² may be a potential predictor for an increased risk of macrosomia. This exploratory threshold may serve as a preliminary reference for risk communication and weight management counseling. Enhanced antenatal monitoring by obstetricians and pediatricians may be considered for those with a BMI above this threshold. However, our findings require external validation in independent cohorts.

## Data Availability

The raw data supporting the conclusions of this article will be made available by the authors, without undue reservation.
