# Supplementary material for: Prepregnancy body mass index and risk of macrosomia after fresh embryo transfer: a retrospective cohort study with exploratory threshold analysis
Source: Front Endocrinol (Lausanne). 2026 Jul 15;17:1884067. doi: 10.3389/fendo.2026.1884067 (PMC13414946; doi:10.3389/fendo.2026.1884067)
Supplement: Supplementary file 1 [file Table1.docx]

Supplementary Table S1: Univariate analysis of impact factors on birthweight and gestational age (n=2195).

| **Exposure** | **Change in birthweight (g)** | | **Change in gestational age (w)** | |
| --- | --- | --- | --- | --- |
|  | **β(95%CI)** | ***P* value** | **β(95%CI)** | ***P* value** |
| **Maternal age (y)** | 0.43 (-3.64, 4.50) | 0.8364 | -0.02 (-0.04, -0.01) | **0.0035** |
| **Paternal age (y)** | -0.03 (-3.49, 3.43) | 0.9883 | -0.02 (-0.03, -0.01) | **0.0028** |
| **Maternal BMI (kg/m^2^)** | 15.99 (9.00, 22.99) | **<0.0001** | -0.03 (-0.05, -0.00) | **0.0372** |
| 18.5-24.9kg/m^2^ (n=1559) | Ref |  | Ref |  |
| < 18.5kg/m^2^ (n=170) | -98.03 (-178.18, -17.89) | **0.0166** | 0.08 (-0.19, 0.35) | 0.5491 |
| 25-29.9kg/m^2^ (n=357) | 68.10 (9.88, 126.32) | **0.0220** | -0.10 (-0.29, 0.10) | 0.3398 |
| ≥30kg/m^2^ (n=109) | 82.59 (10.37, 166.56) | **0.0175** | -0.53 (-1.01, -0.04) | **0.0331** |
| **AMH (ng/ml)** | 0.88 (-8.95, 10.70) | 0.8610 | 0.02 (-0.01, 0.06) | 0.1574 |
| **Type of infertility** |  |  |  |  |
| Primary | Ref |  | Ref |  |
| Secondary | 41.60 (9.08, 74.13) | **0.0122** | -0.15 (-0.27, -0.02) | **0.0196** |
| **Infertility duration (y)** | -7.68 (-13.44, -1.92) | **0.0090** | -0.04 (-0.06, -0.01) | **0.0014** |
| **Infertility cause** |  |  |  |  |
| Female | Ref |  | Ref |  |
| Male | -16.15 (-57.72, 25.42) | 0.4465 | 0.04 (-0.12, 0.20) | 0.6244 |
| Mixed | -13.63 (-70.14, 42.89) | 0.6365 | -0.14 (-0.35, 0.07) | 0.1876 |
| Unexplained | -41.53 (-109.16, 26.09) | 0.2287 | -0.26 (-0.52, 0.01) | 0.0552 |
| **Prior gonadotropin cycle** | -2.68 (-32.19, 26.83) | 0.8589 | -0.09 (-0.21, 0.03) | 0.1564 |
| **COS protocols** |  |  |  |  |
| GnRH-a long protocol | Ref |  | Ref |  |
| Antagonist protocol | -41.66 (-76.24, -7.08) | **0.0183** | -0.15 (-0.28, -0.01) | **0.0310** |
| **Dosage of gonadotropins (IU)** | 0.00 (-0.01, 0.02) | 0.5079 | -0.00 (-0.00, 0.00) | 0.2925 |
| **Stimulation duration (days)** | 5.48 (-2.08, 13.03) | 0.1555 | -0.00 (-0.03, 0.03) | 0.8806 |
| **E2 level on hCG day (pg/ml)** | -0.02 (-0.04, -0.01) | **0.0130** | 0.00 (-0.00, 0.00) | 0.7751 |
| **P level on hCG day (ng/ml)** | -96.27 (-162.77, -29.77) | **0.0046** | -0.23 (-0.46, 0.00) | **0.0550** |
| **Number of oocytes retrieved** | 4.66 (0.31, 9.00) | **0.0357** | 0.02 (0.00, 0.03) | **0.0391** |
| **Fertilization method** |  |  |  |  |
| IVF | Ref |  | Ref |  |
| ICSI | -13.71 (-50.27, 22.85) | 0.4624 | 0.02 (-0.12, 0.16) | 0.7612 |
| IVF+ICSI | -45.46 (-212.98, 122.05) | 0.5948 | -0.07 (-0.72, 0.59) | 0.8380 |
| **Stage of embryos transferred** |  |  |  |  |
| D3 | Ref |  | Ref |  |
| D4 | 21.72 (-47.20, 90.65) | 0.5367 | -0.28 (-0.52, -0.04) | 0.0243 |
| D5 | 47.62 (11.06, 84.18) | **0.0107** | -0.00 (-0.14, 0.13) | 0.9508 |
| **Number of embryos transferred** |  |  |  |  |
| 1 | Ref |  | Ref |  |
| 2 | -23.00 (-56.48, 10.49) | 0.1783 | 0.05 (-0.08, 0.18) | 0.4276 |
| 3 | 80.42 (-333.34, 494.18) | 0.7033 | 0.57 (-1.00, 2.14) | 0.4773 |
| **Endometrial thickness (mm)** | 12.52 (0.85, 24.19) | **0.0356** | 0.05 (0.02, 0.09) | **0.0064** |
| **Endometrial type** |  |  |  |  |
| A | Ref |  | Ref |  |
| A-B | 88.48 (-98.85, 275.82) | 0.3547 | 0.16 (-0.48, 0.79) | 0.6273 |
| B | -8.97 (-162.59, 144.65) | 0.9089 | -0.18 (-0.70, 0.34) | 0.4947 |
| B-C | 15.40 (-136.85, 167.66) | 0.8428 | -0.15 (-0.67, 0.36) | 0.5586 |
| C | 14.97 (-150.45, 180.39) | 0.8592 | -0.21 (-0.77, 0.35) | 0.4694 |
| **Newborn sex** |  |  |  |  |
| Male | Ref |  | Ref |  |
| Female | -75.60 (-108.07, -43.14) | **<0.0001** | 0.33 (0.21, 0.46) | **<0.0001** |
| **Gestational age (w)** | 162.00 (153.75, 170.26) | **<0.0001** | --- | --- |

CI, confidence interval; Ref, reference group.

Supplementary Table S2: Tests for trend across maternal pre-pregnancy BMI categories for birthweight, Z-score, macrosomia, and LGA.

| **BMI category** | **Birthweight (g)**  **Adjust mean (95%CI)** | **Z-score**  **Adjust mean (95%CI)** | **Macrosomia**  **Adjust OR (95% CI)** | **LGA**  **Adjust OR (95% CI)** |
| --- | --- | --- | --- | --- |
| 18.5-24.9 kg/m² | 3268.79 (3241.93, 3295.65) | 0.17 (0.10, 0.24) | Ref* | Ref* |
| < 18.5 kg/m² | 3212.39 (3127.58, 3297.20) | 0.03 (-0.19, 0.25) | 0.47 (0.14, 1.57) | 0.15 (0.02, 1.09) |
| 25-29.9 kg/m² | 3346.56 (3288.45, 3404.67) | 0.41 (0.26, 0.56) | 2.25 (1.29, 3.93) | 1.54 (0.89, 2.66) |
| ≥30 kg/m² | 3495.02 (3330.93, 3659.11) | 0.80 (0.38, 1.22) | 4.56 (2.26, 9.22) | 2.85 (0.86, 9.42) |
| **P for trend** | **0.006** | **0.001** | **<0.001** | 0.0868 |

Analyses were adjusted for prior gonadotropin cycle, COS protocols, type of infertility, infertility duration, dosage of gonadotropins, E2 and P level on hCG day, number of oocytes retrieved, endometrial thickness, endometrial type, stage of embryos transferred, pregnancy complication, newborn sex and gestational age.

For continuous variables (birthweight and Z-score), the adjusted means for the reference group are presented in the corresponding columns. For categorical variables (macrosomia and LGA), the reference group OR is set to Ref. *Ref indicates the reference group (BMI 18.5-24.9 kg/m²).

Supplementary Table S3: Sensitivity analysis: neonatal outcomes across maternal pre-pregnancy BMI categories.

| **Characteristics** | **Adjusted Ⅰ β / OR**  **(95% CI)** | ***P* value** | **AdjustedⅡβ / OR**  **(95% CI)** | ***P* value** | **Adjusted Ⅲ β / OR**  **(95% CI)** | ***P* value** |
| --- | --- | --- | --- | --- | --- | --- |
| **Birthweight (g)** | | | | | | |
| 18.5-24.9 kg/m^2^ | Ref |  | Ref |  | Ref |  |
| < 18.5 kg/m^2^ | -48.99 (-138.44, 40.46) | 0.2833 | -49.01 (-137.74, 41.06) | 0.2844 | -47.29 (-121.11, 50.46) | 0.2722 |
| 25-29.9 kg/m^2^ | **78.88 (13.76, 144.00)** | **0.0177** | **78.92 (12.36, 144.10)** | **0.0176** | **78.94 (13.89, 144.10)** | **0.0191** |
| ≥30 kg/m^2^ | **231.50 (63.33, 399.68)** | **0.0071** | **232.61 (62.35, 398.48)** | **0.0078** | **232.44 (63.39, 398.98)** | **0.0056** |
| **Z-score** | | | | | | |
| 18.5-24.9 kg/m^2^ | Ref |  | Ref |  | Ref |  |
| < 18.5 kg/m^2^ | -0.14 (-0.37, 0.09) | 0.2260 | -0.12 (-0.39, 0.11) | 0.2260 | -0.15 (-0.39, 0.12) | 0.2178 |
| 25-29.9 kg/m^2^ | **0.24 (0.08, 0.41)** | **0.0042** | **0.23 (0.05, 0.44)** | **0.0044** | **0.25 (0.10, 0.48)** | **0.0031** |
| ≥30 kg/m^2^ | **0.63 (0.21, 1.06)** | **0.0037** | **0.62 (0.19, 1.08)** | **0.0039** | **0.64 (0.23, 1.09)** | **0.0030** |
| **Fetal macrosomia (≥4000g)** | | | | | | |
| 18.5-24.9 kg/m^2^ | Ref |  | Ref |  | Ref |  |
| < 18.5 kg/m^2^ | 0.47 (0.14, 1.57) | 0.2180 | 0.46 (0.13, 1.58) | 0.2178 | 0.48 (0.16, 1.60) | 0.190 |
| 25-29.9 kg/m^2^ | **2.25 (1.29, 3.93)** | **0.0044** | **2.23 (1.27, 3.96)** | **0.0041** | **2.29 (1.30, 3.99)** | **0.0038** |
| ≥30 kg/m^2^ | **4.56 (2.26, 9.22)** | **<0.0001** | **4.54 (2.22, 9.29)** | **<0.0001** | **4.77 (2.39, 10.01)** | **<0.0001** |
| **LGA (>90^th^ percentile)** | | | | | | |
| 18.5-24.9 kg/m^2^ | Ref |  | Ref |  | Ref |  |
| < 18.5 kg/m^2^ | 0.15 (0.02, 1.09) | 0.0603 | 0.13 (0.02, 1.13) | 0.0612 | 0.16 (0.02, 1.10) | 0.0781 |
| 25-29.9 kg/m^2^ | 1.54 (0.89, 2.66) | 0.1256 | 1.52 (0.88, 2.69) | 0.1248 | 1.55 (0.90, 2.81) | 0.1367 |
| ≥30 kg/m^2^ | 2.85 (0.86, 9.42) | 0.0868 | 2.78 (0.82, 9.57) | 0.0761 | 2.89 (0.89, 9.76) | 0.0568 |

**Adjust I model** adjust for prior gonadotropin cycle, COS protocols, type of infertility, infertility duration, dosage of gonadotropins, E2 and P level on hCG day, number of oocytes retrieved, endometrial thickness, endometrial type, stage of embryos transferred, pregnancy complication, newborn sex and gestational age.

**In adjustedⅡmodel,** in addition to the variables adjusted for in Model 1, the number of embryos transferred was also included.

**Adjust Ⅲ model** adjust for prior gonadotropin cycle, COS protocols, type of infertility, infertility duration, dosage of gonadotropins, E2 and P level on hCG day, number of oocytes retrieved, endometrial thickness, endometrial type, stage of embryos transferred, hypertensive disorders complicating pregnancy, GDM, intrahepatic cholestasis of pregnancy, newborn sex and gestational age.

OR, odds ratio; CI, confidence interval; Ref, reference group.

**P* value <0.05, ***P* value <0.01.

Supplementary Table S4: Baseline characteristics after Propensity Score Matching (PSM).

| **Variables** | **Group1：BMI ≤26.22 kg/m2**  **n=226** | **Group2：BMI > 26.22 kg/m2**  **n=226** | **P value** | **SMD** |
| --- | --- | --- | --- | --- |
| **Maternal age (y)** | 31.94 ± 3.83 | 32.16 ± 4.11 | 0.5542 | 0.056 |
| **Paternal age (y)** | 33.31 ± 4.59 | 33.41 ± 4.82 | 0.8261 | 0.021 |
| **Type of infertility** |  |  | 0.0884 | 0.169 |
| Primary | 91 (40.3%) | 110 (48.7%) |  |  |
| Secondary | 135 (59.7%) | 116 (51.3%) |  |  |
| **Infertility duration** | 3.52 ± 2.99 | 4.15 ± 3.25 | 0.0315 | 0.202 |
| **Prior gonadotropin cycle** |  |  | 0.3622 | 0.075 |
| 1 | 195 (86.3%) | 189 (83.6%) |  |  |
| 2 | 25 (11.1%) | 33 (14.6%) |  |  |
| 3 | 6 (2.7%) | 4 (1.7%) |  |  |
| **COS protocols** |  |  | 0.7663 | 0.073 |
| GnRH-a long protocol | 147 (65%) | 139 (61.5%) |  |  |
| Antagonist protocol | 79 (35%) | 87 (38.5%) |  |  |
| **Dosage of gonadotropins (IU)** | 2210.90 ± 1240.16 | 2488.05 ± 1119.02 | 0.0130 | 0.235 |
| **E2 level on hCG day (pg/ml)** | 2569.43 ± 1146.69 | 2554.30 ± 1234.74 | 0.8926 | 0.013 |
| **P level on hCG day (ng/ml)** | 0.62 ± 0.41 | 0.62 ± 0.30 | 0.9076 | 0.000 |
| **Number of oocytes retrieved** | 9.85 ± 3.94 | 10.42 ± 3.72 | 0.1198 | 0.148 |
| **Stage of embryos transferred** |  |  | 0.0080 | 0.044 |
| D3 | 105 (46.5%) | 110 (48.7%) |  |  |
| D4 | 15 (6.6%) | 33 (14.6%) |  |  |
| D5 | 106 (46.9%) | 83 (36.7%) |  |  |
| **Endometrial thickness (mm)** | 10.26 ± 1.85 | 10.61 ± 2.01 | 0.0567 | 0.179 |
| **Newborn sex** |  |  | 1.0000 | 0.000 |
| Male | 123 (54.4%) | 123 (54.4%) |  |  |
| Female | 103 (45.6%) | 103 (45.6%) |  |  |
